# Genetic Variation in the TAS2R38 Bitter Taste Receptor and Gastric Cancer Risk in Koreans

**DOI:** 10.1038/srep26904

**Published:** 2016-06-01

**Authors:** Jeong-Hwa Choi, Jeonghee Lee, Il Ju Choi, Young-Woo Kim, Keun Won Ryu, Jeongseon Kim

**Affiliations:** 1 Molecular Epidemiology Branch, Division of Cancer Epidemiology and Prevention,National Cancer Center, 323 Ilsan-ro, Ilsandong-gu, Goyang-si,Gyonggi-do,410-769,Korea.; 2 Center for Gastric Cancer, National Cancer Center, 323 Ilsan-ro, Ilsandong-gu, Goyang-si, Gyonggi-do, 410-769, Korea.

## Abstract

The human *TAS2R38* gene encodes a bitter taste receptor that regulates the bitterness perception and differentiation of ingested nutritional/poisonous compounds in the oral cavity and gastrointestinal tract. *TAS2R38* gene variants are associated with alterations in individual sensitivity to bitter taste and food intake; hence, these genetic variants may modify the risk for diet-related diseases, including cancer. However, little is known about the association between *TAS2R38* polymorphisms and gastric cancer susceptibility. The present case-control study examined the influence of *TAS2R38* polymorphisms on food intake and determined whether they predict gastric cancer risk in Koreans. A total of 1,580 subjects, including 449 gastric cancer cases, were genotyped for *TAS2R38* A49P, V262A, I296V and diplotypes. Dietary data were analysed to determine the total consumption of energy, fibre, vegetables, fruits, sweets, fats, alcohol and cigarettes. *TAS2R38* diplotype was not associated with food, alcohol or cigarette consumption, either independent or dependent of gastric cancer phenotype. However, the PAV/AVI diplotype significantly increased gastric cancer risk (adjusted odds ratio: 1.513; 95% confidence interval: 1.148–1.994) independent of dietary intake. Findings suggest that *TAS2R38* may be associated with the risk for gastric cancer in Koreans, although the *TAS2R38* diplotype did not influence dietary intake.

Taste sensitivity plays a central role in individual dietary behaviour. Differential taste sensitivity leads to differing attraction to a variety of foods; hence, it may influence consumption^1^. Human taste consists of five major categories (sweet, sour, salty, umami and bitter), of which bitterness is a key determinant for the acceptance and/or rejection of foods, such as bitter-tasting vegetables, alcohol and sweets^2^,^3^,^4^. For this reason, it has been hypothesized that differential bitter taste sensitivity may contribute to the risk for diet-related health outcomes, such as cancer^5^,^6^.

Human bitterness perception is mediated by signalling of transmembrane G protein-coupled receptors encoded by *type 2 bitter-taste receptor* (*TAS2R*) genes. Approximately 25 types of functional *TAS2Rs* are located on chromosomes 5, 7 and 12 and are expressed in various organs, including the brain, oral cavity, lung, pancreas and gastrointestinal mucosa^7^,^8^,^9^. Chemosensing by TAS2Rs differentiates bitter compounds as well as beneficial and noxious substances in the diet as a checkpoint^2^,^10^. In particular, TAS2Rs in the gastrointestinal tract initiate the subsequent process of digestion and nutrient absorption. TAS2Rs also trigger protective responses, such as vomiting and excretion, to remove toxic chemicals by activating hormonal and neuronal cascades systems^8^,^11^.

Among the *TAS2R* family, *TAS2R38* gene is the most intensively studied genetic locus for bitter taste perception. Three variants, A49P (145G > C, rs713598), V262A (785T > C, rs1726866) and I296V (886A > G, rs10246939), in the *TAS2R38* gene are associated with bitter taste sensitivity as tested with 6-n-propylthiouracil (PROP) and phenylthiocarbamide (PTC)^12^,^13^. The *TAS2R38* PAV/PAV diplotype is sensitive to PTC/PROP (taster), but the AVI/AVI diplotype rarely responds to thiourea (N-C=S) moiety-containing chemicals (non-taster). Earlier studies demonstrated that *TAS2R38* diplotype is associated with the consumption of bitter foods, cruciferous vegetables (high in a group of thiourea-containing compounds), and alcohol as well as blood folate concentrations^5^,^9^,^14^,^15^,^16^. Therefore, few studies have examined the association between *TAS2R38* genetic variation and disease risk, especially pertaining to cancer, but the findings have been inconclusive^5^,^17^,^18^.

Gastric cancer is one of the common malignancies globally, and the highest incidence rates are found in Eastern Asia, Japan, China, Mongolia and Korea^19^,^20^,^21^. Although the overall incidence and annual mortality of gastric cancer are continuously declining regionally, gastric cancer remains the second most common type of cancer (30,847 new case incidence in 2012), and the third most common reason for cancer-related death in Korea (crude and age-standardized mortality rates in 2012: 18.6 and 11.2 per 100,000)^22^. Diet is a crucial environmental risk factor in gastric cancer aetiology. Abundant intake of vegetables, fruits and fibres reduces the risk for gastric cancer^23^,^24^,^25^, whereas a low folate intake^26^ and a high consumption of red meat and sodium elevate the risk^27^,^28^,^29^,^30^. Epidemiological evidence suggests that various socio-economical and/or psychological factors determine food choice and consumption in individuals^31^,^32^,^33^; however, studies have inadequately addressed the corresponding biological reasons and their association with disease risk. Given the taste sensitivity-diet-disease risk hypothesis, differential taste sensitivity due to *TAS2R38* genetic variants may influence dietary intake and thus modify the risk for gastric cancer. However, to the best of our knowledge, no study has examined the *TAS2R38*-diet association in gastric cancer risk.

This study aimed to examine whether genetic variations in *TAS2R38* influence the consumption of food, alcohol and tobacco in Koreans. The study also investigated whether *TAS2R38* variants are associated with the risk for gastric cancer either dependent or independent of dietary intake.

## Results

### Study population characteristics

Table 1 presents the characteristics of the study participants by gastric cancer phenotype. The subjects with gastric cancer were more likely to be older, male, drinkers and smokers but less likely to engage in regular physical activity compared with the controls (p < 0.001 for all variables except alcohol drinking; p = 0.004 for alcohol drinking behaviour). *Helicobacter pylori* infection was significantly more common among subjects with gastric cancer (p < 0.001). These variables might be associated with gastric cancer risk and were therefore included in subsequent statistical analyses that considered potential confounders. Subjects exhibited significant differences in the dietary intake of the examined foods depending on gastric cancer phenotype. Controls consumed more dietary fibre (p < 0.001), dark green vegetables (p = 0.007), all fruits (p < 0.001) and citrus fruits (p < 0.001) than cases.

### Distribution of *TAS2R38* genetic variants

The distribution of three genotypes and diplotypes of *TAS2R38* is summarized in Table 2. For all three *TAS2R38* variants, the heterozygous genotype was most prevalent. The PA A49P genotype was present in 54.1% and 46.0% of case and control, respectively. The V262A and I296V variants were in complete linkage disequilibrium (*r*^2^ = 1.0), and the prevalence of heterozygous genotypes in both loci were 54.5% for cases and 46.0% for controls. Three out of eight hypothetically possible *TAS2R38* haplotypes were observed in the study population. The most common haplotypes were PAV and AVI, and the combination of these haplotypes (PAV/PAV, PAV/AVI and AVI/AVI) covered most diplotypes observed in the study population. The AAV haplotype was detected in only two subjects with gastric cancer. For this reason, subjects with a diplotype containing an AAV allele (AVI/AAV) were excluded from all statistical analyses. Diplotype is known to depict the interaction between single genetic variation and haplotypes in individuals’ diploid genome and therefore affords greater power in discriminating phenotypic effects^12^,^34^. Thus, the current study focused more on the effect of diplotype than of each single genetic variant. The chi-square tests revealed significant differences in the distribution of *TAS2R38* genotype and diplotype depending on gastric cancer phenotype (Table 2).

### Association between *TAS2R38* diplotype and dietary intake

Table 3 summarizes the mean consumption of selected food groups, alcohol and cigarettes for each major *TAS2R38* diplotype. Total energy, dietary fibre, all vegetables, cruciferous vegetables, dark green vegetables, non-starchy vegetables, all fruits, citrus fruits, sweets, fat-foods and alcohol consumption as well as tobacco smoking were examined. However, no significant differences in those variables were noted among *TAS2R38* diplotypes. When the subjects were stratified based on gastric cancer phenotype, again, *TAS2R38* diplotype did not predict dietary or alcohol intake or cigarette smoking (data not shown).

### Association between *TAS2R38* diplotype and gastric cancer risk

Statistical analyses provided evidence that *TAS2R38* genetic variants are clearly associated with gastric cancer risk, although the variants did not influence dietary intake (Table 4). All three heterozygous genotypes and the PAV/AVI diplotype increased the risk for gastric cancer. The prevalence of PAV/AVI was 54.1% for cases and 46.0% for controls, thus generating an odds ratio (OR) of 1.392 (95% confidence interval (CI): 1.089–1.780). A logistic regression model that considered potential confounders also supported the increased risk for gastric cancer with PAV/AVI diplotype (OR: 1.513; 95% CI: 1.148–1.994).

## Discussion

Genetic variants in human TAS2R38 bitter taste receptor alter individual sensitivity to bitter taste and food intake. Therefore, these variants may be further associated with diet-related disease risk. The present study examined whether *TAS2R38* genetic variants influence dietary intake and whether they are associated with the risk of gastric cancer in Koreans.

The findings showed that the *TAS2R38* diplotype did not predict the dietary intake of the examined foods, alcohol consumption or tobacco smoking, regardless of gastric cancer phenotype. A few earlier studies examined the association between *TAS2R38* diplotype and food and alcohol intake and smoking behaviour; however, the results are inconclusive^17^,^35^,^36^. This inconsistent association between *TAS2R38* diplotypes and dietary food intake might stem from the use of natural and artificial condiments in the study population^17^. Although cruciferous vegetables constitute a large proportion of the vegetables consumed by Koreans, the majority include Kimchi and/or other pickled dishes with a large amount of seasoning, including sodium, garlic, hot pepper, ginger, vinegar, and fish sauce. These strongly flavoured condiments may mask the bitter taste of cruciferous and other vegetables, and therefore, the *TAS2R38* diplotype may not be associated with the dietary intake of such vegetables. Thus, continuous intake of foods containing large amounts of sugar and fat may also impact the sensitivity to sweet and fatty food as well as to fruits, which may have caused the insignificant difference in the intake of these foods among *TAS2R38* diplotype groups^3^,^36^. Furthermore, increased frequency of dining out and the use of artificial flavour enhancers, such as monosodium glutamate, may mask taste and alter the sensitivity to the native tastes of foods. Finally, the relatively small size of the study population may have limited the potential associations between *TAS2R38* diplotypes and dietary food intake (The power of the general linear models was relatively low across the examined dietary variables. See Supplementary Table S1).

**Figure 1:**
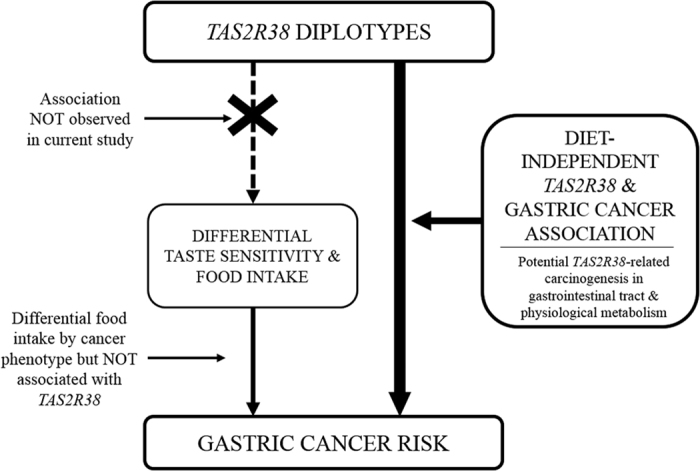
Simplified diagram of the hypothesized (dotted arrow) and observed (solid arrow) association between the *TAS2R38* gene and gastric cancer risk in the present study.

Although *TAS2R38* diplotype was not associated with food intake, *TAS2R38* genetic variants and diplotype predicted gastric cancer risk. This diet-independent *TAS2R38*-cancer association may suggest that genetic variation in *TAS2R38* involved in gastric carcinogenesis not simply via altered food intake but through other potential mechanisms (Fig. 1).

Earlier studies hypothesized that individuals with PAV/PAV diplotype (tasters) may avoid the bitter taste of cruciferous vegetables and may have a greater risk for cancer compared with the AVI/AVI group (non-tasters). However, studies have reported a trend toward an increased risk for colorectal cancer independent of dietary food intake in the AVI/AVI group, not the PAV/PAV group^17^,^18^. The precise mechanism for this phenomenon has not yet been verified. However, one diet-independent hypothesis implicates *TAS2R38* diplotype as a biomarker for gastrointestinal function^18^. The variant protein may give rise to the insufficient ability to neutralize and expel potentially harmful chemicals from the gastrointestinal tract. Therefore, the AVI/AVI diplotype may increase the risk for colorectal cancer and could be a biomarker for gastrointestinal function^7^. This biomarker-related hypothesis possibly supports the diet-independent association between TAS2R38 and gastric cancer risk in the current study^18^,^37^.

Unlike previous studies, our data suggested that the heterozygous *TAS2R38* diplotype (PAV/AVI), not the homozygous AVI/AVI genotype, increased the risk for gastric cancer. A previous study demonstrated that the TAS2R38 receptor encoded by the PAV/PAV variant was only activated by bitter compounds containing a thiourea moiety; other varieties of bitter chemicals minimally activated the protein^37^. The TAS2R38 protein encoded by the AVI/AVI variant rarely responds to PTC and PROP. However, AVI transcript expression was clearly evident, and individuals with the homozygous AVI variant react to other bitter tastes as strongly as individuals with the homozygous PAV variant^37^. This observation may support the notion that the AVI variant not only conveys impaired receptor activity but also potentially mediates the independent reaction to other unknown bitter chemicals^37^. Consistent with these results, both homozygous diplotype receptors (PAV/PAV or AVI/AVI) may be fully capable of responding to each agonist and may thus possess a greater ability to initiate protective mechanisms against potentially toxic chemicals in the gastrointestinal tract. In contrast, compared with homozygotes, the PAV/AVI heterozygous protein may be less active in sensing agonist molecules. Therefore, the gastrointestinal system of individuals harbouring the PAV/AVI diplotype may experience prolonged exposure to potentially carcinogenic chemicals and be at a higher risk for gastric cancer. Findings from studies on chemo-sensitivity and *TAS2R38* diplotype possibly support this hypothesis. Individuals with the PAV/AVI diplotype generally exhibit intermediate sensitivity to bitter-tasting molecules^5^,^37^. Furthermore, allele-specific mRNA expression of the PAV/AVI diplotype was evident, and the expression level of each allele was reduced compared with the PAV and AVI homozygous diplotypes^37^.

Additionally, because gastric carcinogenesis is a multifactorial process, alterations in other TAS2R38-related physiological metabolism processes due to genetic polymorphism may be associated with gastric carcinogenesis. For example, *TAS2R* mRNA expression has been observed in various types of blood leukocytes, which may suggest TAS2Rs contribute to the cellular immune response by differentiating foodborne substances^38^. TAS2R38 protein in ciliated epithelial cells was reported to regulate innate immunity via the production of nitric oxide^39^, which is critical for the initiation and progression of gastric cancer^40^. Therefore, alterations in immune responses and/or nitric oxide production related to *TAS2R38* variants may modify gastric cancer risk if TAS2R38 mediates such defence mechanisms in the gastrointestinal mucosal epithelia. In addition, although the precise mechanism is not fully understood, the TAS2R38 protein functions in energy metabolism and glucose homeostasis^7^,^35^,^41^. The decisive components in these metabolic pathways, including insulin, insulin-like growth factor (IGF), IGF-binding protein and insulin resistance, are also involved in regulating cancer cell proliferation and apoptosis^42^. Therefore, the association between *TAS2R38* variants and these factors may be related to predisposition to gastric cancer. However, because little evidence is available to support those putative correlations, larger epidemiological studies and further investigations are required to verify the underlying mechanisms for the TAS2R38-gastric cancer relationship.

The results of the present study suggest interesting associations surrounding *TAS2R38* bitter taste receptor gene and gastric cancer risk; however, some limitations exist. The findings were derived from a relatively small Korean population, and a limited amount of literature is available to conjecture the association between *TAS2R38* and the gastric carcinogenic mechanism. Although we investigated a myriad of foods and consumer goods, other foods and genes linked to *TAS2R38* may contribute to gastric cancer susceptibility. Despite these limitations, this study is worthy of consideration given that TAS2R38 is centred in human diet behaviour and physiological metabolism. Furthermore, this is the first *TAS2R38* study that covers the full repertoire of genetic variants, dietary intake and clinical phenotype in understanding gastric cancer aetiology.

In summary, the PAV/AVI diplotype of the *TAS2R38* gene increased the risk for gastric cancer, but genetic variation did not influence dietary intake. *TAS2R38* may play a decisive role in gastric carcinogenesis in Koreans.

## Methods

### Subjects

This case-control study was part of a gastric cancer research project at the National Cancer Center (NCC), Korea. The details of the study population were previously described elsewhere^23^,^43^. Participants were recruited at the NCC Hospital between March 2011 and December 2014. Cases were clinically diagnosed by gastroenterology specialists as having early gastric cancer that was histologically confirmed by endoscopic biopsy at the Center for Gastric Cancer. Patients who had diabetes mellitus, severe systemic or mental disease, a history of any other cancer within the past five years or advanced gastric cancer were excluded from the study. Controls were enrolled from a health screening examination (a benefit program of the National Health Insurance) at the Center for Cancer Prevention and Detection in the same hospital. A total of 1,710 subjects (500 gastric cancer cases and 1,210 controls) agreed to participate in the study. In total, 1,585 individuals provided blood samples and were genotyped. The risk for gastric cancer related to *TAS2R38* was estimated from these participants (n = 1,580; 449 gastric cancer cases and 1,131 controls) after excluding subjects whose genotype was not determined (n = 5). Of these subjects, individuals who did not provide dietary intake data (n = 45) or whose total energy intake was <500 kcal or >5,000 kcal (n = 11) were excluded from the study population due to the implausibility of the data. Therefore, the remaining 1,524 participants (1,101 controls and 423 cases) were analysed to determine the influence of *TAS2R38* variants on dietary intake. All participants joined the research voluntarily, and written informed consent was obtained prior to study commencement. All the study protocols were approved by the Institutional Review Board (IRB) of the NCC (IRB Number: NCCNCS-11-148), and all actual procedures involved in current study were conducted in accordance with the guidelines and regulations of the IRB, NCC.

### Data Collection and Dietary Intake Analyses

Participants were requested to complete a self-administered questionnaire designed to collect information on demographics (e.g., age and gender), lifestyle (e.g., cigarette smoking, alcohol consumption and regular physical activity) and medical history. Anthropometric data (e.g., height and weight) were obtained using the InBody 370 (Biospace, Seoul, Korea). Body mass index was computed as weight in kilograms divided by the square of height in meters.

A validated food frequency questionnaire (FFQ) covering 106 food items was used to evaluate dietary intake^44^. The participants were asked to verify the frequency and portion size of each of the food items in the FFQ. Nine degrees of frequency (never or rarely, once a month, two or three times a month, once or twice a week, three or four times a week, five or six times a week, once a day, twice a day, or three times a day) and three portion sizes (small, medium, or large) are included in the FFQ to evaluate food intake over the last year. Dietary intake was evaluated using CAN-PRO 4.0 (Computer Aided Nutritional Analysis Program, The Korean Nutrition Society, Seoul, Korea). The dietary intake of the following nine classes of foods was of particular interest in the current study^17^: 1) total dietary fibre, 2) vegetables (all), 3) cruciferous vegetables (white radish, radish leaves, mustard, mustard leaves, napa cabbage, broccoli, cabbage and others), 4) dark green vegetables (curled mallow, chicory, pumpkin leaf, pine leaf, sweet potato vines, hot pepper, hot pepper leaf, perilla leaf, field dropwort, angelica, water parsley, chives, lettuce, iceberg lettuce, celery, spinach, mugwort, crown daisy, taro vine and others), 5) non-starchy vegetables (all vegetables excluding potato, sweet potato, soybean and green bean), 6) fruits (all), 7) citrus fruits (mandarin, cumquat, orange and orange juice), 8) sweets (honey, candy, sugar, syrup, chocolate, caramel, jam, sherbet, ice cream and soda)^3^ and 9) fat-foods (margarine, butter, beef fat, sesame oil, coffee creamer and soybean oil)^45^. Additionally, the consumption of alcohol (beer, hard liquor, Korean spirits, Korean rice wine, wine and fruit liquor) and tobacco (cigarettes/day), which were previously reported to be associated with taste receptor gene variants, were also investigated^35^,^36^.

### Genotyping and Diplotype Computation

An Axiom^®^ Exome 319 Array (Affymetrix, Santa Clara, CA, USA) containing 318,983 loci of single nucleotide polymorphisms was utilized to determine the *TAS2R38* genotypes of subjects. The call rate for A49P, V262A and I296V *TAS2R38* variants was >95% in both the case and control groups, and all three variants were in Hardy-Weinberg equilibrium in the controls. The *TAS2R38* diplotypes were computed using FAMHAP based on maximum-likelihood estimates, which were generated using the expectation-maximization algorithm (http://famhap.meb.uni-bonn.de/)^46^,^47^.

### Statistical Analyses

Chi-square tests and Student’s t-tests were applied to compare categorical and continuous variables, respectively, by gastric cancer phenotype. Variables for dietary consumption were adjusted for total energy intake using Willett’s residual method^48^. Analysis of variance (ANOVA) was used to evaluate the difference in food intake among the major three diplotypes of *TAS2R38*. Two ANOVA models were established, without (crude) or with adjustments for confounding factors. Logistic regression models without (crude) or with adjustments for potential confounders were applied to determine the ORs and 95% CIs for the associations between *TAS2R38* genetic variants and gastric cancer risk. All the statistical analyses were performed using SAS version 9.3 (SAS Institute Inc., Cary, NC, USA). All reported p-values were two-tailed at the 95% confidence level.

## Additional Information

**How to cite this article**: Choi, J.-H. *et al*. Genetic Variation in the TAS2R38 Bitter Taste Receptor and Gastric Cancer Risk in Koreans. *Sci. Rep*. **6**, 26904; doi: 10.1038/srep26904 (2016).

## Supplementary Material

Supplementary Information

## Figures and Tables

**Table 1:** Descriptive data of study subjects by gastric cancer phenotype.

	Total (N = 1,580)	Case (N = 449)	Control (N = 1,131)	p*
General characteristics				
Number of participants (%)				
Male	832 (52.6)	296 (65.9)	536 (47.3)	<0.001
Female	748 (47.3)	153 (34.0)	595 (52.6)	
Age (years)	53.0 ± 9.31	55.4 ± 10.7	52.1 ± 8.4	<0.001
Body mass index (kg/m^2^)	23.7 ± 3.04	23.6 ± 3.2	23.7 ± 2.9	0.342
Smoking behaviour (%)				<0.001
Non-smoker	815 (51.5)	170 (37.8)	645 (57.0)	
Ex-smoker	416 (26.3)	131 (29.1)	285 (25.2)	
Current smoker	313 (19.8)	131 (29.1)	182 (16.0)	
Missing	36 (2.2)	17 (3.7)	19 (1.6)	
Alcohol drinking behaviour (%)				0.004
Current drinker	916 (57.9)	256 (57.0)	660 (58.3)	
Ex-drinker	123 (7.7)	46 (10.2)	77 (6.8)	
Non-drinker	505 (31.9)	130 (28.9)	375 (33.1)	
Missing	36 (2.2)	17 (3.7)	19 (1.6)	
Regular exercise (%)				<0.001
Yes	746 (47.2)	154 (34.3)	592 (52.3)	
No	793 (50.1)	279 (62.1)	514 (45.4)	
Missing	41 (2.5)	16 (3.5)	25 (2.2)	
*Helicobacter pylori* infection (%)				<0.001
Negative	489 (30.9)	38 (8.4)	451 (39.8)	
Positive	1091 (69.0)	411 (91.5)	680 (60.1)	
Dietary intake				
Energy (kcal/day)	1850.7 ± 638.4	2038.1 ± 694.2	1778.7 ± 600.5	<0.001
Fibre (g/day)	20.4 ± 6.7	19.4 ± 6.5	20.8 ± 6.7	<0.001
Vegetables (g/day)	382.4 ± 194	374.3 ± 203.3	385.6 ± 190.3	0.070
Cruciferous	183.1 ± 118.1	196.4 ± 135.9	178 ± 110.1	0.198
Dark green	41.2 ± 38.3	38.4 ± 36.8	42.3 ± 38.9	0.007
Non-starchy	335.8 ± 177.9	331.4 ± 187.7	337.6 ± 174.0	0.120
Fruits (g/day)	182.1 ± 201.6	135.4 ± 166.9	199.9 ± 210.8	<0.001
Citrus	39.2 ± 55.3	30.7 ± 44.5	42.2 ± 58.4	<0.001
Sweets (g/day)	30.1 ± 63.2	33.5 ± 91.6	28.8 ± 48.2	0.831
Fat-foods (g/day)	4.7 ± 4.8	4.6 ± 5.4	4.7 ± 4.6	0.348

Numbers in parentheses are percentages; all other data are presented as the mean ± standard deviation. Variables for dietary intake were adjusted for energy intake. ^*^P-values denote the difference between cases and controls at the 95% confidence level.

**Table 2:** Distribution of the three *TAS2R38* variants and the diplotypes.

Total (N = 1,580)	Case (%)		Control (%)		p*
	449	(28.4)	1,131	(71.6)	
A49P					
PP	138	(30.7)	412	(36.4)	0.015
PA	243	(54.1)	521	(46.0)	
AA	68	(15.1)	198	(17.5)	
V262A					
AA	138	(30.7)	412	(36.4)	0.009
AV	245	(54.5)	521	(46.0)	
VV	66	(14.7)	198	(17.5)	
I296V					
VV	138	(30.7)	412	(36.4)	0.009
VI	245	(54.5)	521	(46.0)	
II	66	(14.7)	198	(17.5)	
Diplotype					
PAV/PAV	138	(30.7)	412	(36.4)	0.012
PAV/AVI	243	(54.1)	521	(46.0)	
AVI/AVI	66	(14.7)	198	(17.5)	
AVI/AAV	2	(0.4)	–	–	

^*^P-values from chi-square tests.

**Table 3:** Mean consumption of select foods, alcohol and tobacco stratified by *TAS2R38* diplotype.

	PAV/PAV	PAV/AVI	AVI/AVI	p_1_†	p_2_‡
	N = 530 (128, 402)*	N = 736 (232, 504)	N = 256 (61, 195)		
Energy (kcal/day)	1845.1 ± 649.3	1867.1 ± 630.6	1812.5 ± 640.3	0.280	0.223
Fibre (g/day)	20.6 ± 6.4	20.5 ± 6.9	20.1 ± 6.4	0.677	0.435
Vegetables (g/day)	387.9 ± 186.6	382.8 ± 198.1	371.1 ± 197.7	0.363	0.187
Cruciferous	186.5 ± 116.3	184.6 ± 120.2	172.0 ± 115.9	0.198	0.124
Dark green	42.6 ± 36.3	41.3 ± 41.9	38.2 ± 30.9	0.458	0.245
Non-starchy	342.3 ± 171.6	336.1 ± 182.0	322.4 ± 178.9	0.208	0.099
Fruits (g/day)	184.0 ± 203.7	180.2 ± 199.9	183.6 ± 203.5	0.350	0.197
Citrus	43.9 ± 63.2	37.2 ± 52.1	35.0 ± 45.8	0.243	0.266
Sweets (g/day)	29.0 ± 48.8	31.4 ± 76.6	28.7 ± 45.2	0.771	0.879
Fat-food (g/day)	4.6 ± 4.8	4.5 ± 4.5	5.1 ± 5.7	0.402	0.644
Alcohol (g/day)§	15.9 ± 21.9	19.4 ± 28.6	25.4 ± 47.2	0.138	0.336
Tobacco (cigarettes/day)§	16.0 ± 8.8	16.3 ± 8.5	17.0 ± 9.7	0.611	0.513

Dietary variables are presented as the energy-adjusted mean ± standard deviation. Subjects with AVI/AAV diplotype were excluded from the analyses because of limited numbers (n = 2).

^*^Number of subjects (case, control).

^†,‡^Mean intake of subjects with each diplotype was compared without adjustments (p_1_) or after adjusting for sex, age, body mass index, smoking and drinking status, regular exercise and *Helicobacter pylori* infection (p_2_) at the 95% confidence level.

^§^Mean alcohol and tobacco consumption was computed from group of subjects only ex- and current drinkers and smokers.

**Table 4:** *TAS2R38* variants and diplotypes and the association with gastric cancer risk.

	Model 1 OR (95% CI)*	*P*	Model 2 OR (95% CI)†	*P*
A49P				
PP	1.000 (reference)		1.000 (reference)	
PA	1.392 (1.089–1.780)	0.005	1.515 (1.149–1.996)	0.001
AA	1.025 (0.733–1.435)	0.361	0.983 (0.676–1.429)	0.190
V262A				
AA	1.000 (reference)		1.000 (reference)	
AV	1.404 (1.098–1.794)	0.003	1.525 (1.157–2.009)	0.001
VV	0.995 (0.709–1.396)	0.261	0.957 (0.656–1.395)	0.140
I296V				
VV	1.000 (reference)		1.000 (reference)	
VI	1.404 (1.098–1.794)	0.003	1.525 (1.157–2.009)	0.001
II	0.995 (0.709–1.396)	0.261	0.957 (0.656–1.395)	0.140
Diplotype				
PAV/PAV	1.000 (reference)		1.000 (reference)^‡^	
PAV/AVI	1.392 (1.089–1.780)	0.004	1.513 (1.148–1.994)	0.001
AVI/AVI	0.995 (0.709–1.396)	0.272	0.956 (0.656–1.394)	0.145

Subjects with AVI/AAV diplotype were excluded from the analyses because of limited numbers (n = 2).

^*^Model 1: crude OR and 95% CI.

^†^Model 2: ORs computed after adjusting for sex, age, body mass index, smoking and drinking status, regular exercise and *Helicobacter pylori* infection (please refer to Supplementary Table S2 for the effect of potential confounders). OR = odds ratio; CI = confidence interval.
