# Supplementary material for: Genetic Variation in the TAS2R38 Bitter Taste Receptor and Gastric Cancer Risk in Koreans
Source: Sci Rep. 2016 Jun 1;6:26904. doi: 10.1038/srep26904 (PMC4887993; doi:10.1038/srep26904)
Supplement: Supplementary Information [file srep26904-s1.pdf]

Genetic Variation in the TAS2R38 Bitter Taste Receptor and Gastric Cancer Risk in Koreans

Jeong-Hwa Choi[1], Jeonghee Lee[1], Il Ju Choi[2], Young-Woo Kim[2], Keun Won Ryu[2] and Jeongseon Kim[1*]

[1]Molecular Epidemiology Branch, Division of Cancer Epidemiology and Prevention, [2]Center for Gastric Cancer, National Cancer Center 323 Ilsan-ro, Ilsandong-gu, Goyang-si, Gyonggi-do, 410-769, Korea.

[*]Corresponding Author: Jeongseon Kim, jskim@ncc.re.kr

Supplemental Table S1. The results of the power analysis for each dietary variable

Variables	Power
Energy	0.277
Fibre	0.113
Vegetables	0.228
Cruciferous	0.344
Dark green	0.184
Non-starchy	0.334
Fruits	0.234
Citrus	0.305
Sweets	0.091
Fat-food	0.208
Alcohol	0.409
Tobacco	0.131

Supplemental Table S2. The effect of potential confounders in logistic regression models for association between the *TAS2R38* diplotype and gastric cancer risk

	Effect	Odds Ratio (95% Confidence Interval)	p-value
Gender	Male	Reference (1.000)	
	Female	0.715 (0.484 - 1.055)	0.091
Age	Year	1.044 (1.029 - 1.059)	<.0001
Smoking status	Never	Reference (1.000)	
	Former	1.140 (0.761 - 1.708)	0.123
	Current	2.129 (1.405 - 3.227)	<.0001
Drinking status	Never	Reference (1.000)	
	Former	1.406 (0.902 - 2.192)	0.187
	Current	1.094 (0.810 - 1.478)	0.639
Regular exercise	Yes	Reference (1.000)	
	No	2.184 (1.694 - 2.815)	<.0001
Helicobacter pylori infection	Negative	Reference (1.000)	
	Positive	6.651 (4.605 - 9.605)	<.0001
Body mass index	Kg/m^2	0.961 (0.921 - 1.002)	0.064
